# A Clinicopathological Study of Odontogenic Cysts and Tumors in Hamadan, Iran

**Published:** 2014-12

**Authors:** Fahimeh Baghaei, Massoumeh Zargaran, Hamidreza Najmi, Abbas Moghimbeigi

**Affiliations:** a Dept. of Oral and Maxillofacial Pathology, School of Dentistry, Hamadan University of Medical Sciences, Hamadan, Iran.; b Dental Research Center, Dept. of Oral and Maxillofacial Pathology, School of Dentistry, Hamadan University of Medical Sciences Hamadan, Iran.; c Dentist, Arak, Iran.; d Dept. of Biostatistics and Epidemiology, School of Public Health, Hamadan University of Medical Sciences, Hamadan, Iran.

**Keywords:** Odontogenic cyst, Odontogenic tumor, Oral lesions, Prevalence

## Abstract

**Statement of the Problem:** Odontogenic cysts and tumors are the most frequent osseous destructive lesions of the jaws; however, there is little information regarding the relative frequency of these lesions among the Iranian population.

**Purpose:** The purpose of this study was to determine the distribution of clinically and histologically- diagnosed odontogenic cysts and tumors during a period of 13 years in Hamadan, and also its correlation with age, gender, and the site of the lesion.

**Materials and Method:** A descriptive cross-sectional study was performed on 413 oral and maxillofacial specimens during 1996 to 2008.The age and the gender of patients, as well as the site of lesion were recorded. The data were analyzed using SPSS software.

**Results:** Totally, 70 specimens were recorded as odontogenic cysts and 11 specimens were diagnosed as odontogenic tumors. The most frequent odontogenic cysts were dentigerous cysts (27.2%), followed by radicular cysts (18.6%) and odontogenic keratocysts (18.6%). In addition, cysts were more frequent in male than female individuals. Ameloblastoma was the most frequent odontogenic tumor (64%).

**Conclusion:** Odontogenic cysts were in correlation with age, gender and location. These results showed that dentigerous cyst and odontogenic keratocyst were more frequent than other studies. More investigations should be performed to determine the frequency of odontogenic tumors in Iran.

## Introduction

The human Jaws are considered as the common site for the presence of a wide range of cysts and tumors. Some of them, such as odontogenic cysts and tumors, are originated from tissue remnants of the tooth forming apparatus or are the result of inflammation. Moreover, some of these lesions have shown neoplastic alterations or aggressive clinical behavior.[1-2] Therefore, surgically-excluded tissues (biopsies) should be investigated by pathologists for the relevant and appropriate treatments.

Previous studies declared that the odontogenic cysts and tumors have different epidemiological prevalence. In a study performed in Mexico, the authors reported that the periapical cysts, dentigerous cysts and the odontogenic keratocysts (OKCs) were the most detected cysts.[3]

Bataineh et al.[4] showed the most common odontogenic cysts were radicular and dentigerous cysts, respectively.

The prevalence of odontogenic cysts was studies and  reported to be a male to female ratio of 1.86:1 and a mandibular to maxillary prevalence ratio of 3:1. The most diagnosed cysts were radicular cysts, dentigerous cysts and OKCs respectively.[5]Other studies showed that radicular cyst, dentigerous cyst and OKC exhibited the highest prevalence.[6-7]

A study reported that 10.4% of all oral samples were odontogenic cysts. Radicular cysts and dentigerous cysts were the two most common lesions.[8]A research performed in Nigeria scrutinized  that ameloblastoma and adenomatoid tumors were the most common odontogenic tumors.[9]Ameloblastoma was reported to be the most common tumor of mandiblein a research; while the odontoma was placed in the fourth rank.[10]

In a research enrolled in Brazil, odontoma and ameloblastoma were recorded as the most frequent odontogenic tumors.[11] Other studies showed that 80.1% of odontogenic tumors were ameloblastoma,[12] while odontogenic tumors comprised 1.78% of all jaw lesions.[13]A study  carried out in California showed that odontogenic tumors constituted about 1.2% of all jaw tumors.[14] A study surveyed the children and adolescents of Libya and reported that the relative frequency of benign odontogenic tumors was 16.4%.[15]

Despite the importance of this issue, little inclusive data are available regarding the frequency of odontogenic cysts and tumors in Iran, and particularly the province of the Hamadan. The aim of this study was to determine the frequency of odontogenic cysts and tumors concerning the age, gender distribution and the site of the lesions in Hamadan University of Medical Sciences. 

## Materials and Method

In this retrospective cross-sectional study, we investigated the records related to the patients referred to the pathology centers of University of Medical Sciences of Hamadan, from 1996 to 2009.

We investigated all oral and jaw lesions from the pathology reports and we recorded all the information regarding age, gender, the site of the lesion and the registered histopathological diagnosis. The samples (cases) with incomplete information and indefinite diagnosis were excluded. All histologically-diagnosed odontogenic cysts and tumors were appraised in terms of frequency, age, sex, and their anatomical distribution.

Statistical analysis was performed using SPSS software (15th edition). Odontogenic cysts and tumors were compared considering gender and age using χ2 and Fisher’s exact tests. The level of significance was adopted less than 0.05. 

## Results

From 413 patients recruited in this study, 147 (35.6%) were men, 229 (55.4%) were women and the gender of 37 patients (9%) was unrecorded. Out of 413 cases, 70 cases (16.9%) were odontogenic cysts, 11 cases (2.7%) were odontogenic tumors, and 332 cases (80.4%) were detected as other le-sions (Table 1). The average age of patients with odontogenic cysts and tumors were 27.88 and 23.09 years respectively, and it was 35.71 years in patients diagnosed with other lesions.

**Table 1:** Frequency distribution of oral lesions based on their site.

** Variable type**		**Frequency**	**Percentage**
Type of lesion	Odontogenic cyst	70	16.9
	Odontogenic tumor	11	2.7
	Other lesions	332	80.4
Total		413	100
Site of lesion	Maxilla	54	13.1
	Mandible	57	13.9
	Soft tissue	302	73
Total		413	100

Table 2 indicates that the diagnosis of odontogenic cyst was more frequent in men (55.9%) than in women (44.1%); however, the diagnosis of odontogenic tumors was significantly less prevalent in men (36.4%) than in women (63.4%).

**Table 2:** Frequency distribution of oral lesions based on gender.

**Gender** **Type of lesion**	**Male**		**Female**		**Total**	** Test χ^ 2 ^**
	**Frequency**	**Percentage**	**Frequency**	**Percentage**		
Odontogenic cysts	38	55.9	30	44.1	68	χ^ 2 ^=9.83 df=2 *p*= 0.007
Odontogenic tumors	4	36.4	7	63.6	11	
Other lesions	105	35.4	192	64.6	297	
Total	147	39.1	229	60.9	376	

As illustrated in Table 3, odontogenic cysts mostly occurred in the third and fourth decades of life (61%) followed by the age >40 years (22%). This table also indicates that odontogenic tumors were more predominant in the age range of 20-40 years (90.9%).

**Table 3:** Frequency distribution of oral lesions based on age.

**Age** **Lesion**	**≤ 20**		**20 < age ≤ 40**		**> 40**		**Fisher’s** **Exact Test**
	**Frequency**	**Percentage**	**Frequency**	**Percentage**	**Frequency**	**Percentage**	
Odontogenic cysts	10	16.9	36	61.0	13	22.0	20.62 df=4 *p*< 0.0001
Odontogenic tumors	1	9.1	10	90.9	0	0	
Other lesions	17	6.3	138	51.3	114	42.4	
Total	28	8.2	184	54.2	127	37.4	

Among 70 reports of odontogenic cysts, dentigerous cyst (27.2%) revealed a higher incidence in men and in the maxilla. According to the Table 4, the prevalence of other odontogenic cysts has correspondingly been the radicular cyst (18.6%, more prevalent in men and the maxilla), OKC (18.6%, more common in men and the mandible), and calcified odontogenic cyst (11.4%, predominantly in women and the maxilla). Ameloblastoma (64%) was the most common odontogenic tumor that showed a higher prevalence in male than female individuals (57.1%); which was also more common in the mandible.

**Table 4:** Frequency distribution of odontogenic cysts and tumors based on incidence

	**Lesion**	**Frequency**	**Percentage**
Odontogenic cysts	Periapical cyst	13	18.6
	Dentigerous cyst	19	27.2
	Odontogenic keratocyst	13	18.6
	Calcifying odontogenic cyst	8	11.4
	Eruption cyst	1	1.4
	Residual cyst	1	1.4
	Nonspecific inflammatory	15	21.4
	Total	70	100
Odontogenic tumors	Ameloblastoma	7	64
	Adenomatoid odontogenic tumor	1	9
	Odontoma	1	9
	Cementoblastoma	1	9
	Odontogenic fibroma	1	9
	Total	11	100

Among 70 odontogenic cysts, 37 cases were located in the maxilla, 21 cases were detected in the mandible and the location of 12 cases was not registered. In all types of the cysts investigated in the present study, the frequency of involvement of the maxilla was higher than the mandible, except the eruption cyst and the OKC in which the frequency was higher in the mandible. In addition, from 11 odontogenic tumors; 2 cases were diagnosed in the maxilla (18.2%) and 9 cases (81.8%) were assessed in the mandible. On the other hand, in many cases, the exact location of the involvement was not registered clearly.  

## Discussion

The total number of 422 samples in the current study was very restricted regarding the population of Hamadan. The prevalence of odontogenic cysts in this study (16.9% of all the biopsies of oral and jaw lesions) was relatively similar to those related to Singapore and Brazil; [7, 16] whilst the reported prevalence of these lesions in Sicily (10.4%) and England (12.8%) was in the lower range. [6, 8] The difference may be attributed to the limited amount of biopsies taken from other lesions compared to the odontogenic cysts taken by oral and maxillofacial surgeons. This will consequently lead to an increase in the proportion of the detected odontogenic cysts, although the pattern of odontogenic cysts may vary in different geographical regions.

In the current study, dentigerous cyst was the most prevalent odontogenic cyst, followed by radicular cyst and OKC in the experimented population. This was in accordance with the findings of Dhanuthai et al.[17] and Ochsenius et al. [18] who reported the dentigerous cyst as the most common cyst in children in Thailand and Chile. Nevertheless, other studies considered the periapical cyst as the most common odontogenic cyst and the dentigerous cyst as the second most prevalent. [3-6, 19-20]

The prevalence of radicular cyst ranged from 37.8% in the study of Sharifian et al. to 84.5% in the review of Tortoric et al. [8, 20] In contrast; our study revealed the radicular cyst was the second most common cyst. One probable reason of this issue might be that maxillofacial surgeons in Hamadan usually do not send the periapical inflammatory tissues associated with the extracted teeth for histological evaluations. Another reason could be attributed to the high percentage of unspecified inflammatory cysts, resulted from the insufficient clinical and radiological information sent to the referred pathologist.

In the present study, the frequency of odontogenic cysts in male individuals was slightly higher than the female cases, which was similar to the other studies.[4-5, 8, 18-19] Inflammatory cysts such as radicular cysts were mostly diagnosed in male patients; since women are probably more concerned to their oral health.In our study, OKC was the second most common odontogenic cyst, however, in most studies; it has been the third most commonly diagnosed cyst. [3-6, 8, 18-21]

Moreover, calcifying odontogenic cyst has shown the high prevalence in contrast to the results of Jones et al.’s study [6]The different results yielded by various studies could be due to the different protocols employed in their studies. For instance, some surveys have been performed only in children or some surveys only have studied the developmental tumors.

Inclusively, this study showed that odontogenic cysts were more common in maxilla than mandible which was in agreement with the results of some studies [6, 8, 19] and in contrast with other studies who reported the same prevalence of odontogenic cysts in both jaws [4] and Meningaud et al.’s study which conveyed higher prevalence in mandible. [5]

According to the results of this study, the peak incidence of odontogenic cysts occurred in the third and fourth decades of life. The study of Cabrini et al. has shown the highest prevalence of oral cysts in the age of 30-50 years. [22] Bataineh et al. [4] reported the third to fifth decades of life as the most common age range; which is virtually the same as the findings of the present study. Odontogenic tumors are uncommon lesions, but their frequency in the oral biopsies was variable regarding the studies conveyed in different countries. [9-15]

According to the pathology reports in the present study, odontogenic tumors included 2.7% of all oral and jaw biopsies. The reports, testified from America continent, confirmed that the prevalence of these lesions was less than 2%. [14] There are also some studies that concluded the similar results to  our study’s, such as Santos et al., [11] Buchner et al. with the prevalence of 1.2%, [14] Fernandes et al. with a prevalence of 1.78% [13] and Ochesenius et al. with a prevalence of 1.29%. [23]

These findings, compared to the results of similar studies enrolled in Africa and other Asian countries, imply that the prevalence of lesions in Iran is lower than the prevalence reported from African regions and other Asian countries. [16, 24-25]

It is noteworthy that in the investigations carried out among population under the age of 20 years; the higher prevalence of odontogenic tumors has been established. The prevalence of these tumors, in young people in Argentina, has been reported 7% [26] and in adolescent of Libyan 16.4%. [15] The difference in the prevalence of odontogenic tumors in different parts of the world denotes that the incidence of these lesions possibly is related to the racial and environmental factors.

Our results revealed that ameloblastoma was the most common odontogenic tumor. Similar findings were reported in the studies accomplished in Asian countries. [10, 16, 27-28] Furthermore, studies performed in Africa disclosed the same results. [12, 25]

It is noteworthy that due to the small number of odontogenic tumors observed in this study, some tumors have not been diagnosed or merely small numbers have been reported. Subsequently, further studies are recommended to find out the relative frequency of odontogenic tumors and cysts in our country. Additionally, the authors believe that patients should be encouraged for periodic clinical and radiological examinations to prompt the treatment of oral and maxillofacial tumors and cysts. Dentists, as the pioneers of dental examinations, should be also responsible in taking the biopsies from the suspicious patients and send them for histopathological examinations. All these issues will enhance the precise diagnosis of odontogenic lesions and would pledge their efficient treatments along with minimizing the related physical, mental and social difficulties. 

## Conclusion

The results of the current study, with its all limitations, displayed that different types of odontogenic cysts and tumors could involve the deliberated population and some of them tended to appear in the certain gender, age and location. Dentigerous cyst and odontogenic keratocyst were more frequent than other studies and ameloblastoma was the most common odontogenic tumor. Considering the few number of odontogenic tumors in this study, further investigations are required to determine the relative frequency of odontogenic tumors in Iran. 
